# Supplementary material for: Clustering algorithm for formations in football games
Source: Sci Rep. 2019 Sep 11;9:13172. doi: 10.1038/s41598-019-48623-1 (PMC6739367; doi:10.1038/s41598-019-48623-1)
Supplement: Supplementary file 1 — Supplementary Material [file 41598_2019_48623_MOESM1_ESM.pdf]

Supplementary Material: Clustering algorithm for formations in football games

Takuma Narizuka[1] and Yoshihiro Yamazaki[2]

[1]Department of Physics, Faculty of Science and Engineering, Chuo University, Bunkyo, Tokyo 112-8551, Japan
[2]Department of Physics, School of Advanced Science and Engineering, Waseda University, Shinjuku, Tokyo 169-8555, Japan

Corresponding author: Takuma Narizuka
E-mail address: pararel@gmail.com

This PDF file includes:
Figure S1. Heat maps (average formations) of five games for 18 teams.

Supplementary Figure

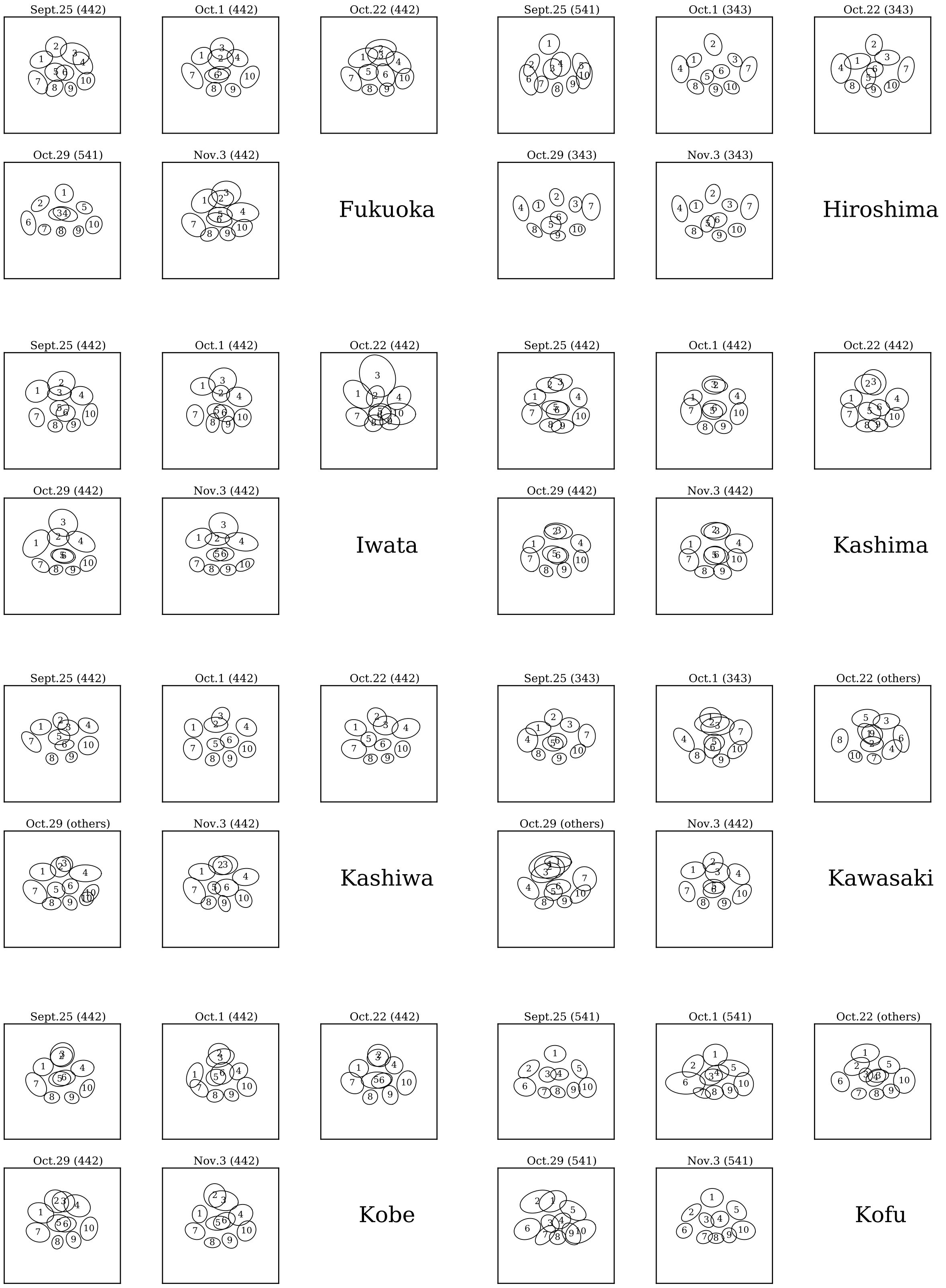

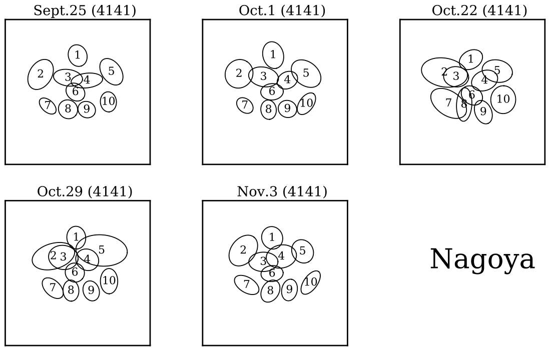
Sept.25 (4141)
Oct.1 (4141)
Oct.22 (4141)
Oct.29 (4141)
Nov.3 (4141)
Nagoya

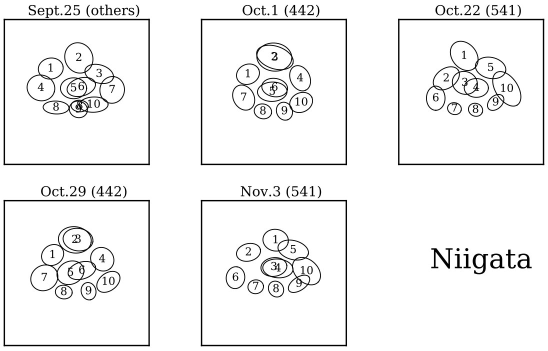
Sept.25 (others)
Oct.1 (442)
Oct.22 (541)
Oct.29 (442)
Nov.3 (541)
Niigata

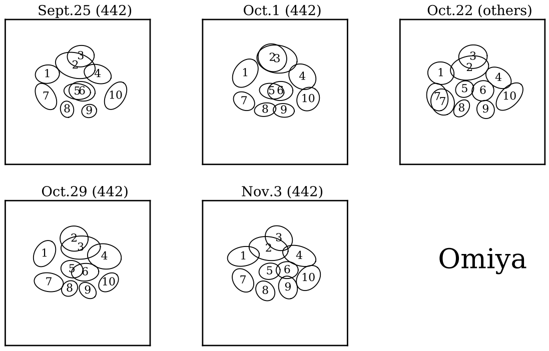
Sept.25 (442)
Oct.1 (442)
Oct.22 (others)
Oct.29 (442)
Nov.3 (442)
Omiya

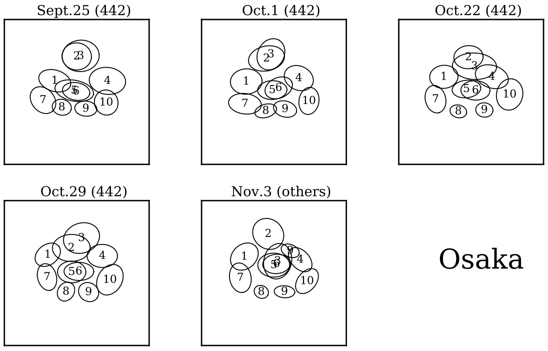
Sept.25 (442)
Oct.1 (442)
Oct.22 (442)
Oct.29 (442)
Nov.3 (others)
Osaka

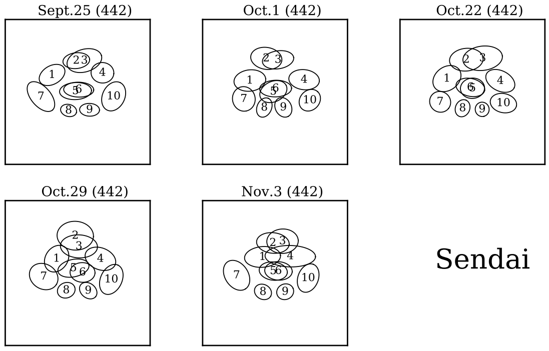
Sept.25 (442)
Oct.1 (442)
Oct.22 (442)
Oct.29 (442)
Nov.3 (442)
Sendai

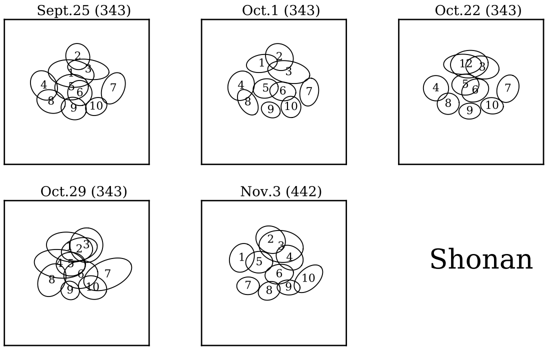
Sept.25 (343)
Oct.1 (343)
Oct.22 (343)
Oct.29 (343)
Nov.3 (442)
Shonan

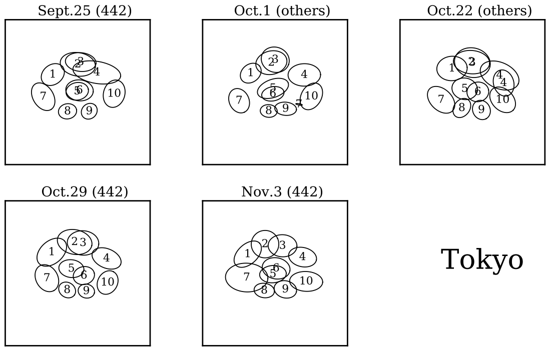
Sept.25 (442)
Oct.1 (others)
Oct.22 (others)
Oct.29 (442)
Nov.3 (442)
Tokyo

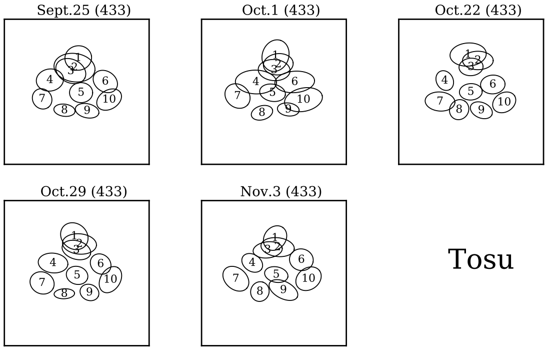
Sept.25 (433)
Oct.1 (433)
Oct.22 (433)
Oct.29 (433)
Nov.3 (433)
Tosu

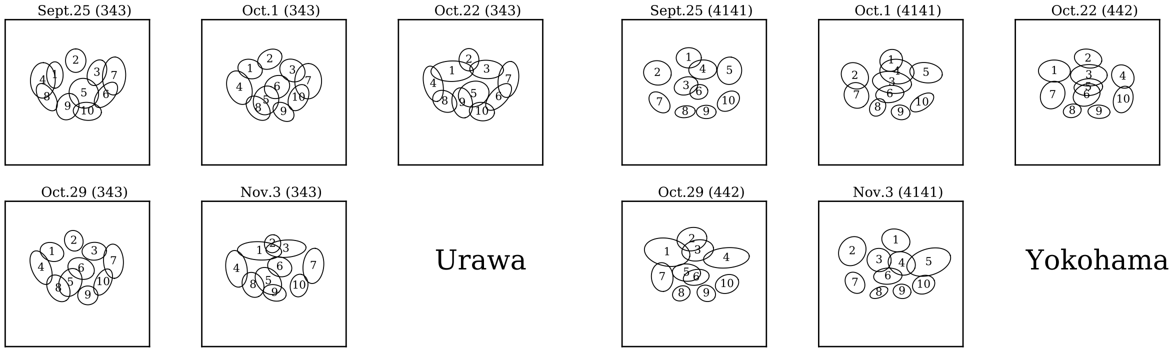

Figure S1. Heat maps (average formations) of five games for 18 teams in the normalized coordinates. The direction of offense is upward in each panel. Each heat map belongs to one of the following five average formations: “442,” “4141,” “433,” “541,” and “343.” The label “(others)” means that player substitutions occurred in the first half of the game, or the average formation could not be identified.
